# Distribution of Domoic Acid in the Digestive Gland of the King Scallop *Pecten maximus*

**DOI:** 10.3390/toxins12060371

**Published:** 2020-06-04

**Authors:** Juan Blanco, Aida Mauríz, Gonzalo Álvarez

**Affiliations:** 1 Centro de Investigacións Mariñas, Xunta de Galicia, Pedras de Corón S/N, 36620 Vilanova de Arousa, Spain; amauriz@cimacoron.org; 2 Facultad de Ciencias del Mar, Departamento de Acuicultura, Universidad Católica del Norte, Larrondo 1281, Coquimbo, Casilla 117, Chile; 3 Centro de Investigación y Desarrollo Tecnológico en Algas (CIDTA), Facultad de Ciencias del Mar, Larrondo 1281, Universidad Católica del Norte, Coquimbo Casilla 117, Chile

**Keywords:** domoic acid, digestive gland, cellular types, accumulation, heterogeneity, toxin distribution, depuration

## Abstract

The king scallop *Pecten maximus* retains the amnesic shellfish poisoning toxin, domoic acid (DA), for a long time. Most of the toxin is accumulated in the digestive gland, but this organ contains several cell types whose contribution to the accumulation of the toxin is unknown. Determining the time-course of the depuration by analyzing whole organs is difficult because the inter-individual variability is high. A sampling method, using biopsies of the digestive gland, has been developed. This method allows for repetitive sampling of the same scallop, but the representativeness of the samples obtained in this way needs to be validated. In this work, we found that the distribution of DA in the digestive gland of the scallops is mostly homogeneous. Only the area closest to the gonad, and especially its outer portion, had a lower concentration than the other ones, probably due to a transfer of the toxin to the intestinal loop. Samples obtained by biopsies can therefore be considered to be representative. Most of the toxin was accumulated in large cells (mostly digestive cells), which could be due to differences during the toxin absorption or to the preferential depuration of the toxin from the small cells (mostly secretory).

## 1. Introduction

In 1987 in Prince Edward Island, Canada, four people died and nearly 100 became intoxicated by the consumption of blue mussels *Mytilus edulis.* The main symptoms of this intoxication were digestive problems and short-term memory loss, which led to the syndrome being named Amnesic Shellfish Poisoning [1,2,3]. Domoic acid (DA), a tricarboxylic amino acid previously isolated from the red alga *Chondria armata* [4], was identified as the responsible agent [5]. The diatom *Nitzschia pungens f. multiseries* (currently *Pseudo-nitzschia multiseries*) was later identified as the producing organism in the area [6]. Since then, this toxin has been recorded in many locations around the world including Europe [7,8,9,10,11], North America [12,13,14,15,16,17], South America [18], Oceania [19], Asia [20,21,22,23,24,25,26] and Africa [27,28,29]. DA, beside its effect on humans, also has severe effects on other marine mammals, such as sea lions [30,31,32,33], sea otters [34], different cetaceans [35,36] and birds [37,38,39]. Consequently, it has a high economic and ecological importance, especially in the marine environment [40].

As the presence of DA in bivalves represents a major risk for the health of consumers, many countries have regulated its maximum allowable level in bivalve molluscs and have established how this compound should be monitored. In most cases, the allowable levels are near those initially established in Canada, which correspond to 20 mg kg^−1^ [41]. The existence of regulation leads to collecting and marketing bans when the allowable levels are surpassed, affecting fishery and aquaculture. The incidence of DA for these economic sectors depends on the capability of the involved bivalve species of accumulating the toxin, which is a species-specific characteristic that depends on the balance between absorption and elimination.

Most commercially important bivalve species depurate DA within hours or a few days, as is the case for mussels *Mytilus edulis* [42,43], *M. californianus* [44], *M. galloprovicialis* [45] and *Perna canaliculus* [19], the oyster *Crassostrea virginica* [46] and the surf clam *Mesodesma donacium* [47], or in a few weeks, as is the case for the scallop *Placopecten magellanicus* [48]. Consequently, they retain the toxin for a short time and the impact on their harvest and commercialization is low. Other species, nevertheless, can retain the toxin for a long time, like the razor clam *Siliqua patula* in [15,49], of which DA binds to some receptors [50], and the king scallop *Pecten maximus* [8,51,52,53,54], which probably lacks a membrane transporter to efficiently excrete it [55].

In Europe, *Pecten maximus* is a commercially important species mainly because of three reasons: (a) it has a high market value; (b) there are large populations in several areas and (c) it is a good species for aquaculture due to its fast growth. However, recurrent blooms of the DA producing diatoms of genus *Pseudo-nitzschia* have led to important distortions in its harvesting and the discouragement of aquaculture efforts because, frequently, the affected scallops do not become safe for consumers before the next *Pseudo-nitzschia* bloom (which, in NW Spain, for example, develops twice a year [56,57]), thus concatenating long closure periods (in some cases, several years) [58]. It has been demonstrated that most of the toxin is accumulated in the non-edible parts of the scallops (mainly in the digestive gland) [53,54,59]. In an attempt to mitigate this problem, the European Union has made an exception to the general way of monitoring bivalves for human consumption, by allowing for the selective evisceration of the most toxic parts prior to sale. This exception only applies to *Pecten maximus* and *P. jacobeus* because of their special DA retention characteristics among bivalves. For these species, the DA concentration in the entire shellfish must be less than 250 and less than 4.6 mg kg^−1^ in each of the edible tissues (gonad and adductor muscle) [60]. Even with this exception, the exploitation of the natural beds is very limited and the aquaculture of *Pecten maximus* has not developed significantly, stressing the need for methods to mitigate the impact of DA. The experimental studies of depuration needed are hampered by the high inter-individual variability in toxin concentration in the scallop. To reduce this variability, for most bivalves a large number of individuals are used, but for the king scallop this strategy is difficult. Due to its large size, it is difficult to include many individuals in the experiments because they would require important facilities. A way to reduce the number of scallops required while maintaining a reduced variability during depuration would be to take repeated measurements from the same scallop. That is not possible with the usual methods of toxin determination, which are destructive, but it can be done by taking biopsies (of the digestive gland, which accumulates more that 90% of the toxin in the scallops), as shown in a previous work [61]. Different parts of the digestive gland might have different concentrations of DA, in which case the samples obtained by biopsies would not be representative of the whole organ, thus making it necessary to evaluate the heterogeneity of the DA distribution in that organ.

The depuration of DA from the king scallops, and especially from their digestive gland, is very slow [53,54]. The mechanisms and routes by which the toxin is eliminated are not known, but it seems that a membrane transporter could be involved [55]. Different cell types could have different membrane transporters, and consequently have different DA depuration capabilities. Even when it is clear that the digestive gland is the main organ in which the toxin is accumulated, nothing is known about how the DA is distributed among both of the different cell types of the tissues involved. The digestive gland is constituted by several cell types whose functions are in some cases under discussion [62]. The two main types are absorptive (digestive) or secretory (basophile) cells. The first ones are in charge of the absorption of particles and their intracellular digestion and are larger than the secretory cells. Secretory cells produce enzymes and secrete them to the digestive lumen [62,63]. It could, therefore, be expected that the larger absorptive cells contain more DA than the smaller secretory ones, unless the transfer from absorptive to secretory cells is high.

**Figure 1:**
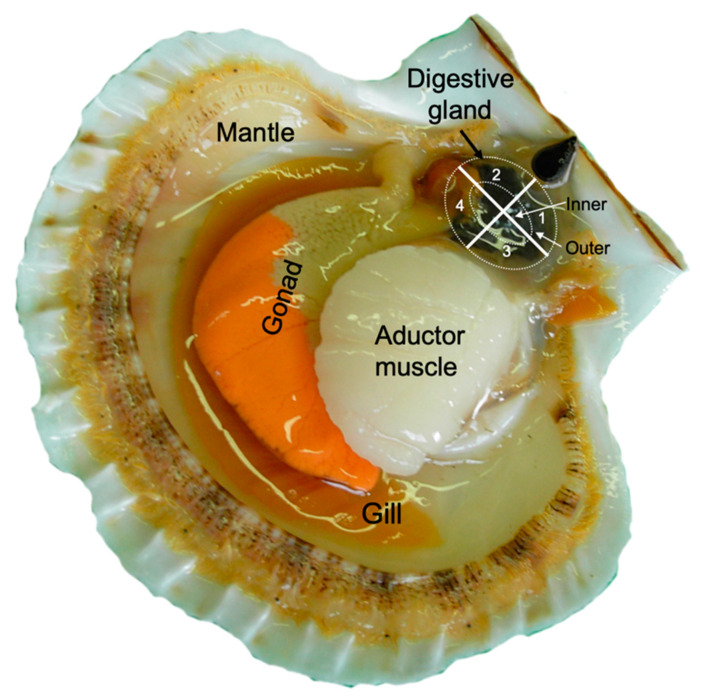
Schematic diagram of a scallop including the sectors (1 to 4) and regions (inner and outer, separated by a dotted line) into which the digestive gland was divided.

In this study the distribution of the DA among different regions (Figure 1), sectors and cellular types of the digestive gland of *Pecten maximus* was determined to (1) evaluate the heterogeneity of the toxin concentration in the digestive gland, to contribute to the understanding and minimization of the variations observed in repeated samples of this organ obtained using biopsies, which could be a non-destructive way of monitoring the depuration process, and (2) gain some information about the actual depuration routes or mechanisms.

## 2. Results

### 2.1. Variability of Toxin Distribution in Different Parts of the Digestive Gland

The variation coefficients estimated from all sectors and regions of each scallop were low–moderate, between 8.5% and 27.1%.

**Figure 2:**
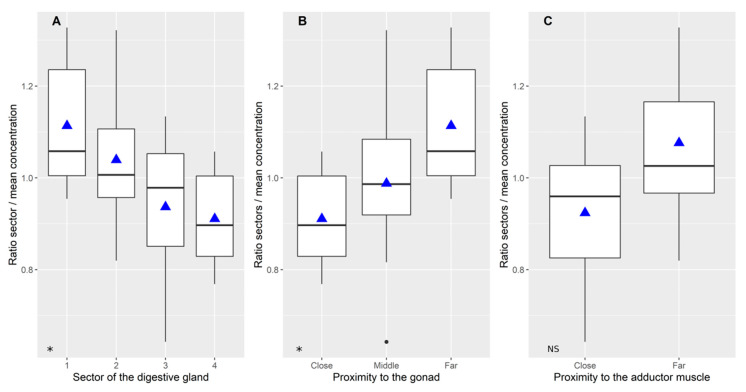
The relative concentration of domoic acid in different areas of the scallop digestive gland: Difference between sectors (**A**); and as a function of the distance to the gonad (**B**); and to the adductor muscle (**C**). The box limits are the quartiles, the middle horizontal line is the median, the vertical lines (whiskers) are the range, the dots are the outliers and the triangles are the means. NS and * indicate the non-significance and significance of the ANOVA, respectively.

Domoic acid was not homogeneously distributed among the four sectors of the digestive gland (Figure 2A, *p* = 0.0159 by a two-way ANOVA, Supplementary Materials Table S1). Sector 1 had the highest relative concentration and sector 4 the lowest. On average, the difference between these two sectors was approximately 20% of the mean. When the effect of the distance to the gonad (Figure 2B) and the adductor muscle (Figure 2C) was examined, the toxin concentration was found to be lower as sector 4 was closer to the gonad (*p* = 0.0385, by a two way ANOVA, Supplementary Materials Table S2), with the range also being approx. 20%. The toxin concentration was lower in the sectors closer to the adductor muscle (3 and 4), but the difference was not statistically significant (*p* = 0.2746 by a two-way ANOVA, Supplementary Materials Table S2).

**Figure 3:**
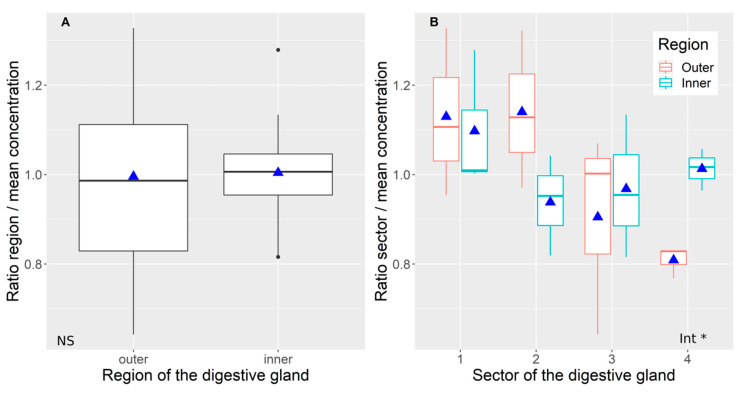
The effect of the part of the digestive gland (inner or outer) on the relative concentration of domoic acid in the whole digestive gland (**A**) and each sector (**B**). Plot details as in Figure 2. NS and Int * indicate the non-significance and significance of the interaction in ANOVA, respectively.

The toxin was, in general, homogeneously distributed between the internal and external regions of the digestive gland (Figure 3A, *p* = 0.8921 in a 2-way ANOVA on the relative concentration, Supplementary Materials Table S1.). The variability, nevertheless, was higher in the outer than in the inner parts (Figure 3A) In two sectors (2 and 4) the concentration in these two parts was different (lower in the outer part in sector 4, and the opposite in sector 2) (Figure 3B), which made the interaction between sector and part (inner-outer) near to the statistical significance (*p* = 0.08, a 2-way ANOVA on the relative concentration, Supplementary Materials Table S1).

### 2.2. Distribution of Domoic Acid between Cellular Types

**Figure 4:**
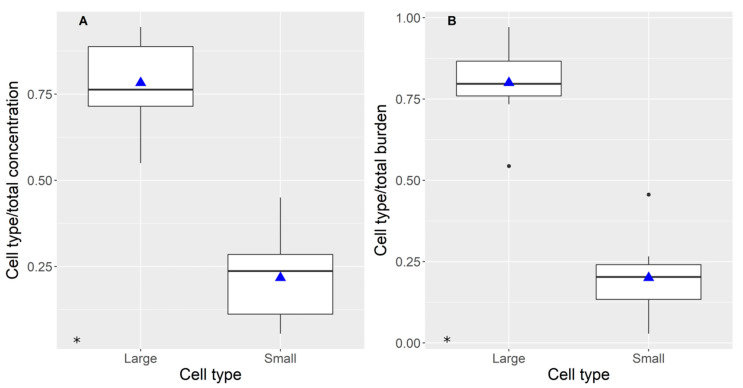
Relative domoic acid concentration (**A**) and burden (**B**) in large and small cells of the digestive gland. Plot details as in Figure 2. * indicates the significance of the ANOVA.

Domoic acid was preferentially accumulated into large (mostly digestive) cells (Figure 4A, *p* = 0.0039 by Wilcoxon paired samples test). The measured concentration of this toxin in small cells (basophilic, mostly secretory) was less than 25% of that in large cells.

The recorded differences in toxin burden (Figure 4B) were higher than those in concentration because the large cells contributed more to the digestive gland weight than the small ones.

## 3. Discussion

The variability of the toxin concentration among areas of the digestive gland of the scallops is moderate-low. It is lower than the usual inter-individual variation in scallops [52,59] and other bivalves [47,64,65]. Most sectors of the digestive gland had very similar concentrations, with the only significant exception being the sector adjacent to the gonad. In addition, in this sector the outer region was shown to have a lower concentration than the inner one. The most likely cause for this difference is the migration of the toxin to the intestinal loop located in the gonad. In previous studies [66], it has been observed that gonadal growth during depuration produces an increase in DA burden in this organ. The origin of the toxin incorporated by the intestinal loop should be the digestive gland, which is where the DA in the scallops is mainly stored. The mechanism involved, nevertheless, is unknown. A possibility could be the reabsorption of the toxin excreted by the digestive gland by the intestinal cells. It has been shown that those cells can significantly absorb different substances [62], but the very low depuration rate of the scallops [53,54] suggests that the amount of DA that could be reabsorbed is very low. In any case, this mechanism would not be expected to affect some parts of the gonad differently. A second possibility could be the transfer of the toxin from the digestive gland cells to the newly generated intestinal ones. Transfers of DA between organs have been documented but are significant only in exceptional situations, such as post-spawning periods [53,66,67]. This mechanism could or could not have an effect on the distribution of DA in the different areas of the digestive gland. The third possibility is that the intestinal loop cells which contain the toxin derive from cells from the digestive gland. If the new cells of the intestinal loop are generated in the nearby area of the digestive gland, a reduction in the toxin concentration in that area would be expected. Intestinal cells are mostly epithelial and the presence of immature cells, as in the digestive gland, has not been observed [62]. It is therefore possible that the intestinal cells derive from immature cells in the digestive gland and consequently inherit the toxin they contain. This would explain the lower DA concentration in the sector closest to the gonad and also the greater reduction in the outer region relative to the inner one, because the cells in this region are the closest to the intestinal loop. The confirmation of this hypothesis requires additional studies.

Taking into account the involved mechanisms proposed and the observed variability, it seems clear that, in general, sampling any part of the digestive gland could be enough to trace the time-course of the toxin accumulation in that organ, which accumulates more than 90% of the DA in the scallops. Non-destructive sampling utilizing biopsies [61], for example, could be used without targeting a precise part of the organ. Nevertheless, avoiding sampling the sector adjacent to the gonad could reduce the variability, especially during periods of intense gonadal growth.

Biopsies could be especially useful for experimental studies of DA depuration in *Pecten maximus* because of its size, commercial value and high inter-individual variation. In most studies of bivalves, the problem of inter-individual variation is usually reduced by using a large number of organisms, which are analysed individually or in pooled samples. Large numbers of scallops are difficult to place in most experimental facilities and this, together with the economic cost, prevents its use in most cases. Sampling the same scallops during the depuration period would reduce the interindividual variability to the variability between biopsies and, from our study, it seems clear that the biopsies could be representative of the whole digestive gland (which contains more than 90% of the toxin in the scallop). For monitoring natural beds, using biopsies could also have some benefits because it would be not necessary to sacrifice the scallops to have a rough estimate of their toxin concentration.

Domoic acid was preferentially retained in large cells, mostly absorptive (digestive cells) [68]. The concentration measured in small cells, mostly secretory and immature cells, was substantially lower than in the digestive ones. In terms of toxin burden, the importance of large cells was even greater, because they are found in a larger amount in the digestive gland (more biomass). The difference in concentration opens two possibilities regarding the methods of depuration not taken into account to date: (a) the secretory cells depurate the DA they have accumulated during the intoxication phase (which could be the case if both cell types accumulate the toxin to the same extent); and (b) DA is transferred from absorptive to secretory cells, which excrete it. Unfortunately, no information about the distribution of DA during the intoxication phase exists and the transfer between cellular types has not been documented, so new studies would be required to shed some light on the subject. However, in our opinion, neither of the two ways is very likely. Firstly, it seems unlikely that absorptive and secretory cells would absorb and accumulate DA during the intoxication at the same rate, because of their main functions. Secondly, it is difficult to explain how the toxin could pass the cellular membrane of the absorptive cells to go to the secretory ones but not to be excreted (the depuration rate is very low [53,54]) or transferred to other tissues [53,54].

The same difference in toxin concentration between cellular types of the digestive gland was found in the mussel *Mytilus galloprovincialis* for okadaic acid. Okadaic acid is a lipophilic toxin [69], which is chemically very different to DA, suggesting that the mechanism underlying the observed differences could be independent of the chemical structure of the toxins.

## 4. Materials and Methods

### 4.1. Biological Material

The scallops used in this work were collected from the Ría de Arousa after a toxic *Pseudo-nitzschia* bloom and maintained for two to four days in running seawater.

### 4.2. Sector Distribution

The digestive glands of three scallops were dissected into four sectors, which were chosen based on their proximity to the adductor muscle (1 and 3) and to the gonad (2 and 4). The external and internal regions of each sector were also separated (Figure 1), taking into account that the first one is expected to be enriched with acini and the second one with primary and secondary ducts. Therefore, eight fractions of each gland were obtained. All inner regions of the dissected sectors contained some gastric tissue, which was not removed because it would have also been sampled in the biopsies.

Each fragment was weighed and extracted with methanol 50% (1:36 *w*/*v*) by homogenization with Ultraturrax (12,000 rpm, 3 min) and centrifuged at 9960× *g* for 25 min. Throughout the extraction process, the samples were kept in an ice bath. A 0.5-mL aliquot of each extract was collected for HPLC analysis.

### 4.3. Cell Type Separations

The dissociation of the digestive gland pieces to obtain the cells was carried out using the method of Giard et al. [70], which involves an enzymatic treatment. The digestive glands from nine scallops were divided into 1–2 mm pieces, in 20 mM, pH 7.4, HEPES buffer, containing 436 mM NaCl, 53 mM MgSO_4_, 10 mM KCl, 10 mM CaCl_2_ and 11 mM glucose. The fragments were washed twice in this medium and then dissociated enzymatically through two 30-min incubations in the previous buffer containing 0.1% pronase under gentle agitation, at room temperature. The cell suspension obtained was filtered through a 100 μm Nylon mesh and the cell types were separated by centrifugation.

After several tests at different centrifugation speeds and checking the separation of cell types by microscope examination, 43.5 and 77× *g* were chosen to precipitate large and small cells, respectively. Thus, the cell suspension was centrifuged for five minutes at 43.5× *g*. The pellet containing the larger cell types (mostly digestive cells) was reserved. The smaller cell types (mainly secretory cells and non-differentiated cells) were then precipitated from the supernatant by centrifugation at 77× *g* for 5 min and the supernatant was discarded.

Each pellet was weighed after centrifugation and careful removal of the supernatant. The DA was extracted by sonication in 50% MeOH (1:10 *w*/*v*) for 10 min in a sonication bath and by centrifugation at 11,136× *g* for 5 min. A 0.5 mL aliquot of each extract was collected for HPLC analysis.

### 4.4. Chromatographic Analysis

The analyses were carried out by HPLC-UV using a Thermo Surveyor PDA and the method of Regueiro et al. [71] with small modifications and omitting the preliminary cleaning steps. A monolithic column Chromolith Performance RP-18 (100 × 3 mm, Merck) was used and the separation was carried out isocratically with ACN: HCOOH: H2O (7:0.2:92.8 *v*/*v*/*v*) as mobile phase, flow 1.2 mL/min and the column temperature was maintained at 30 °C. An injection volume of 20 µL was used. The absorbance was monitored at 242 and 265 nm, corresponding to the peak wavelengths at which DA and tryptophan (its main interfering compound) absorb, respectively. The limit of detection of the method is less than 0.1 µg g^−1^.

### 4.5. Computations and Statistical Analysis

The ratios of the different parts or sectors to the mean were obtained by dividing the toxin concentration in that part or sector by the mean concentration in the digestive gland. The mean concentration in the digestive gland was calculated with the expression ∑C_p_·W_p_/∑W_p_, where C_p_ is the concentration in each piece (part-sector) and W_p_ are the corresponding weights.

All of the statistical analyses were carried out with R [72] and all of the figures were generated with the R package ggplot2 [73].

## Supplementary Materials

The following are available online at https://www.mdpi.com/2072-6651/12/6/371/s1. Table S1: Two-way ANOVA of the DA concentration relative to the mean of the digestive gland as a function of the sector, the inner or outer area and their interaction, Table S2: Two-way ANOVA of the DA concentration relative to the mean of the digestive gland as a function of the distance to the gonad, the distance to the adductor muscle and their interaction.
