# Supplementary material for: Distribution of Domoic Acid in the Digestive Gland of the King Scallop Pecten maximus
Source: Toxins (Basel). 2020 Jun 4;12(6):371. doi: 10.3390/toxins12060371 (PMC7354575; doi:10.3390/toxins12060371)
Supplement: Supplementary file 1 [file toxins-12-00371-s001.pdf]

Supplementary material: Distribution of Domoic Acid in the Digestive Gland of the King Scallop *Pecten maximus*

Juan Blanco, Aida Mauríz and Gonzalo Álvarez

Table S1. Two-way ANOVA of the DA concentration relative to the mean of the digestive gland as a function of the distance to the gonad, the distance to the adductor muscle and their interaction.

Source	Df	Sum Sq	Mean Sq	*F* value	Pr(>F)
sector	1	0.1513	0.15134	6.936	0.0159
ext_int	1	0.0004	0.00041	0.019	0.8921
sector:ext_int	1	0.0713	0.07126	3.266	0.0858
Residuals	20	0.4364	0.02182		

Table S2. Two-way ANOVA of the DA concentration relative to the mean of the digestive gland as a function of the sector, the inner or outer area and their interaction.

Source	Df	Sum Sq	Mean Sq	*F* value	Pr(>F)
sector	1	0.1231	0.12305	4.911	0.0385
ext_int	1	0.0316	0.03162	1.262	0.2746
sector:ext_int	1	0.0035	0.00353	0.141	0.7112
Residuals	20	0.5012	0.02506		
